# Detection of spontaneous pulse using the acceleration signals acquired from CPR feedback sensor in a porcine model of cardiac arrest

**DOI:** 10.1371/journal.pone.0189217

**Published:** 2017-12-08

**Authors:** Liang Wei, Gang Chen, Zhengfei Yang, Tao Yu, Weilun Quan, Yongqin Li

**Affiliations:** 1 School of Biomedical Engineering, Third Military Medical University, Chongqing, the People's Republic of China; 2 Emergency Department, Sun Yat-Sen Memorial Hospital of Sun Yat-Sen University, Guangzhou, Guangdong, the People's Republic of China; 3 ZOLL Medical Corporation, Chelmsford, Massachusetts, United States of America; Universidad del Pais Vasco, SPAIN

## Abstract

**Background:**

Reliable detection of return of spontaneous circulation with minimal interruptions of chest compressions is part of high-quality cardiopulmonary resuscitation (CPR) and routinely done by checking pulsation of carotid arteries. However, manual palpation was time-consuming and unreliable even if performed by expert clinicians. Therefore, automated accurate pulse detection with minimal interruptions of chest compression is highly desirable during cardiac arrest especially in out-of-hospital settings.

**Objective:**

To investigate whether the acceleration (ACC) signals acquired from accelerometer-based CPR feedback sensor can be used to distinguish perfusing rhythm (PR) from pulseless electrical activity (PEA) in a porcine model of cardiac arrest.

**Methods:**

Cardiac arrest was induced in 49 male adult pigs. ECG, arterial blood pressure (ABP) and ACC waveforms were simultaneously recorded during CPR. 3-second segments containing compression-free signals during chest compression pauses were extracted and only those segments with organized rhythm were used for analysis. PR was defined as systolic arterial pressure >60 mmHg and pulse pressure >10 mmHg, while PEA was defined as an organized rhythm that does not meet the above criteria for PR. Peak correlation coefficient (CCp) of the cross-correlation function between pre-processed ECG and ACC, was used to discriminate PR and PEA.

**Results:**

63 PR and 153 PEA were identified from the total of 1025 extracted segments. CCp was significantly higher for PR as compared to PEA (0.440±0.176 vs. 0.067±0.042, *p*<0.01) and highly correlated with ABP (*r* = 0.848, *p*<0.001). The area under the receiver operating characteristic curve, sensitivity, specificity and accuracy were 0.965, 93.6%, 97.5% and 96.7% for the ACC-based automatic spontaneous pulse detection.

**Conclusions:**

In this animal model, the ACC signals acquired from an accelerometer-based CPR feedback sensor can be used to detect the presence of spontaneous pulse with high accuracy.

## Introduction

During cardiopulmonary resuscitation (CPR), chest compression (CC) is frequently interrupted for rhythm analysis and pulse checking [1]. The shock advice algorithms in automated external defibrillators (AEDs) are able to identify shockable rhythms and organized rhythms with high accuracy [2, 3]. On the other hand, current AEDs are still not able to distinguish a perfusing rhythm (PR) from a non-perfusing rhythm, when an organized rhythm is present [4]. To assess pulse presence during CPR, manual palpation is still routinely used. However, the manual palpation was proved to be time-consuming and inaccurate, as well as highly subjective, with a reported sensitivity of 90% and specificity of 55% [5–8] and is only recommended for the experienced healthcare providers [9]. The proportion of time spent on performing CC, has been shown to be associated with defibrillation success rate and survival [10, 11]. However, the manual palpation may take 25 seconds or more in some cases [12, 13]. In addition, the high false detection rate by the manual palpation may decrease the possibility of successful resuscitation by stopping CC for the patient who should be treated by continuous CC.

As the gold standard for circulation detection, invasive measurement of the systolic blood pressure is only feasible at the time of resuscitation in a very small minority of patients in critical care settings [14, 15]. The current Guidelines recommended using capnogram as a decision support tool for return of spontaneous circulation (ROSC) detection [16]. An abrupt increase in end-tidal carbon dioxide level to normal value may serve as an indicator of ROSC [17, 18]. However, capnogram is only available with intubation and suffers low sensitivity in ROSC detection [19]. A false negative may lead to continued CC despite a PR and may increase the probability of unnecessary re-fibrillation [20]. Therefore, automated accurate pulse detection with minimal interruptions of CC is highly desirable during cardiac arrest especially in out-of-hospital settings.

The accelerometer-based CPR feedback pads routinely used to measure compression depth and rate during CPR [21–23], may pick up the force from the chest wall transferred from the heart contractions. In this study, we retrospectively reviewed the acceleration (ACC) signals acquired from accelerometer-based CPR feedback sensor and investigated whether it can be used to distinguish PR from pulseless electrical activity (PEA) in a porcine model of cardiac arrest.

## Materials and methods

The experimental data were retrospectively collected from 49 male adult domestic pigs (pig farm of South China Agricultural University, Guangzhou, China) experienced cardiac arrest and CPR in an effort to investigate different strategies of CPR or post-resuscitation therapies [24, 25]. The ACC signals were initially recorded as feedback to monitor and control the compression rate and depth. Experiments were performed in Laboratory Animal Center of Sun Yat-sen University (Guangzhou, China). All animals received humane care and the experiments were conducted after approval of the Animal Ethics Committee, Sun Yat-sen University (IACUC-DD-15-0503). The protocol was performed according to institutional guidelines.

### Experiment procedure and data collection

Animals were fasted overnight except for free access to water. For all of the pigs, anesthesia was initiated by intramuscular injection of ketamine (20 mg/kg), completed by ear vein injection of sodium pentobarbital with a dose of 30 mg/kg and maintained by additional doses of 8 mg/kg. A cuffed endotracheal tube was advanced into the trachea and animals were mechanically ventilated with a volume-controlled ventilator (model Avs III, T Bird, CA, USA), with a tidal volume of 15 mL/kg, a respiration rate of 12–20 breaths/min, and maintained PetCO_2_ at 35–40 mmHg. A fluid-filled catheter was advanced from the right femoral artery into the thoracic aorta for the measurement of arterial blood pressure (ABP). Fifteen min after baseline measurements, VF was electrically induced by applying a 5 mA alternate current through a pacing catheter in the right ventricle in 44 animals. Conventional 30:2 CPR, was begun after 6, 10, 11 or 12 min of untreated VF. For animals subjected to 6 min of untreated VF, a single 120-J biphasic shock (M/R Series, Zoll Medical Corporation, Chelmsford, MA, USA) was attempted to terminate VF after 2 min of CPR. For animals subjected to 10, 11 and 12 min of untreated VF, the defibrillation shock was delivered after 6 min of CPR. For all of the animals, CC were immediately resumed followed by ECG rhythm analysis within 5 s until confirmation of ROSC. If spontaneous circulation was not restored, CC were continued for another 2 min before defibrillation with 120 J was attempted again. This sequence was repeated for a maximum of 5 cycles. In another 5 animals, asphyxia was induced by clamping of the endotracheal tube after mechanical ventilation was removed. Asphyxial cardiac arrest was defined as an aortic systolic pressure of <30 mmHg. After 5 min of untreated cardiac arrest, CC and ventilation strategy which was same to VF cardiac arrest animals was provided. CPR was continued until ROSC or for 10 min if ROSC was not achieved. If VF occurred during CPR, a single 120-J defibrillation was attempted. For all of the animals, ROSC was defined as an organized cardiac rhythm with a mean arterial pressure (MAP) >60 mmHg, which persisted for an interval of 10 min or more [26].

After the experiment, the animals were returned to their cages with a room temperature maintained at 20°C to 24°C. The animals were allowed free access to food and water and were observed for an additional 96 hrs to assess neurological recovery. At the end of 96 hrs post-resuscitation, the animals were euthanized with an intravenous injection of 150 mg/kg sodium pentobarbital.

ECG and ACC signals were acquired from one-piece, pre-connected electrode with an accelerometer-based CPR sensor (CPR-D-padz, ZOLL Medical Corporation, Chelmsford, MA, USA) that was placed on the surface of the animal’s chest over the heart. ECG and ABP were measured with a multi-parameter patient monitor (Datascope 3000, Datascope Crop. Paramus, NJ, USA) with analog output. ECG, ABP and ACC waveforms were then simultaneously recorded through a data acquisition system supported by Windaq hardware/software (Dataq Instruments Inc., Akron, OH, USA) at a sample rate of 300 Hz.

**Fig 1:**
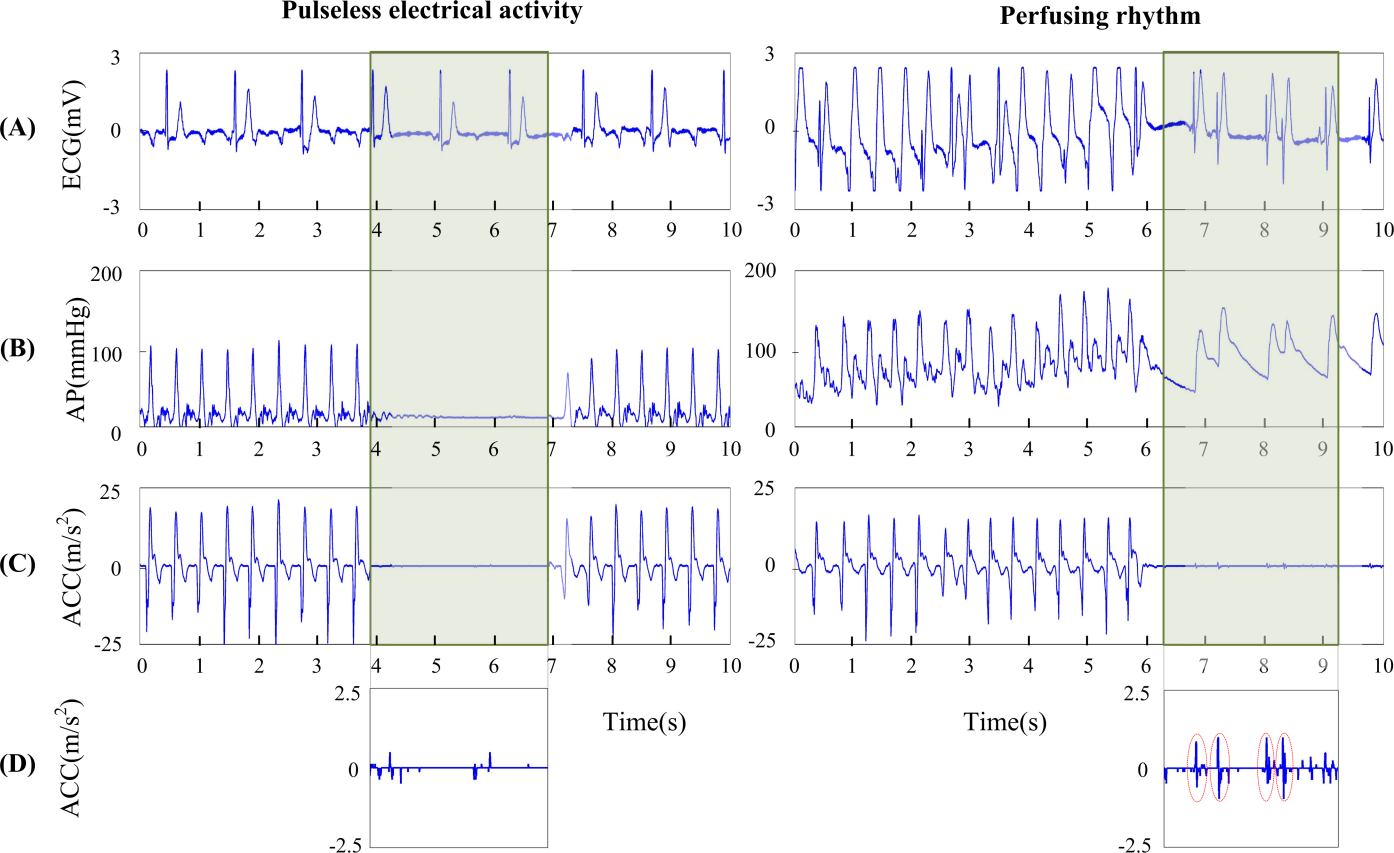
Illustration of ECG, arterial blood pressure (ABP) and acceleration (ACC) signals recorded during cardiopulmonary resuscitation and segments extracted for pulseless electrical activity and perfusing rhythm discrimination.

Data were analyzed through user designed software using Matlab (The MathWorks, Inc., Natick, MA, USA). As CC pauses were between 3 and 5 s during CPR, we extracted 3-s segments of signals at the start of the pauses. Segments with rhythm transitions or defibrillation shocks were excluded from the dataset. As shown in Fig 1, all of the segments contained ECG, ABP and ACC signals. The underlying cardiac rhythms were annotated as organized or disorganized rhythms by an emergency medical doctor through examining the 3-s compression-free ECG signal (shaded area). Organized rhythm was defined as the presence of at least one observational QRS complex within the segment. For organized rhythms, the segments were further classified as PR or PEA by examining the 3-s ABP signal. PR was defined as systolic arterial pressure >60 mmHg and pulse pressure >10 mmHg in the presence of an organized rhythm, while PEA was defined as an organized rhythm that does not meet the above criteria for PR [27, 28].

The annotated dataset was randomized into a training set (32 PR and 74 PEA) and a testing set (31 PR and 79 PEA). PR and PEA segments extracted from same animal were all assigned to either training or testing set. The training set was used to optimize parameters of the ACC-based spontaneous pulse detection algorithm and the testing set was used to assess discriminative ability.

### Spontaneous pulse detection

For each segment, ACC and ECG signals were firstly pre-processed to remove low-frequency interferences such as baseline wander and high-frequency noise. More specifically, ECG was pre-processed with a 4^th^ order Butterworth band-pass filter of 0.2–45 Hz while ACC was pre-processed using a narrow band-pass filter with the cut-off frequencies of 0.5 and 7.5 Hz.

**Fig 2:**
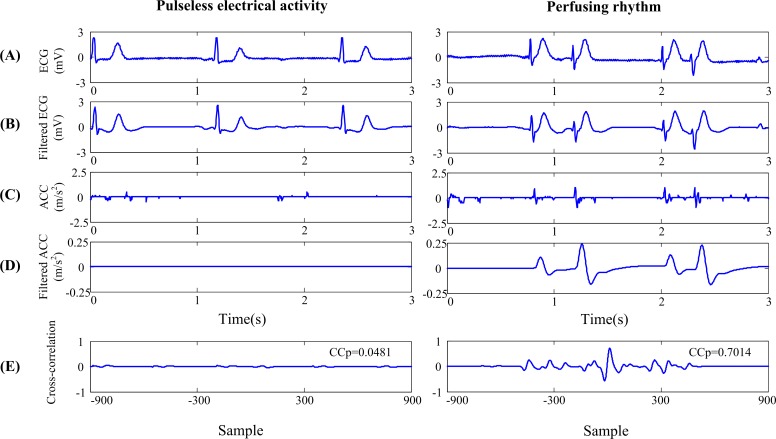
Spontaneous pulse detection based on cross-correlation function between ECG and acceleration (ACC) signals.

The normalized cross-correlation function was then calculated between the filtered ECG and ACC signals (Fig 2) with the following equation:

$$r_{xy}(l)=\frac{1}{\sqrt{r_{xx}r_{yy}}}\sum_{n=1}^{N}x(n)y(n-l),l=0,\pm 1,\pm 2,\cdots \pm N\text{-}1$$ (1)

where $r_{xx}=\sum_{n=1}^{N}x(n)x(n)$ and $r_{yy}=\sum_{n=1}^{N}y(n)y(n)$. *x* and *y* are the filtered ECG and ACC signals with identical sample size of *N* (*N* = 900 in the current study). The peak correlation coefficient (CCp) denoting the maximum of the normalized cross-correlation sequence, was used to quantify the degree of correlation between ECG and ACC and to discriminate PR from PEA.

### Statistical analysis

Data were presented as mean ± standard deviation. MAP, CCp and heart rate (HR) were compared between PR and PEA by two-tailed Student’s *t*-test. The relationship between CCp and MAP was tested with Pearson correlation coefficient. The discriminative ability of CCp was evaluated by determining the area under receiver operating characteristic curve (AUC), sensitivity, specificity, accuracy and compared with that of MAP and HR. Sensitivity is the ability to detect PR. Specificity refers to the ability to correctly classify PEA rhythms. Accuracy is the probability to obtain a correct decision. AUCs were compared by Z-test and proportions were compared by Chi-square test. A *p*<0.05 was considered statistical significant.

## Results

In the 49 pigs weighing between 28 and 43 (36.2±3.1) kg with ECG, ABP and ACC signals all available, 37 animals were successfully resuscitated (33 VF and 4 asphyxia) and 12 animals didn’t achieve ROSC (11 VF and 1 asphyxia). The average duration of CPR was 6.64±2.94 min for VF and 3.87±3.53 min for asphyxial cardiac arrest. A total of 63 PR and 153 PEA rhythms were obtained for analysis according to the inclusion criteria. Among the 63 PR, 56 were extracted from 42 animals experienced VF and 7 were extracted from 4 animals experienced asphyxia. Among the 153 PEA, 78 were extracted from 10 animals experienced VF and 75 were extracted from 5 animals experienced asphyxia.

Fig 1 illustrates the ECG, ABP and ACC waveforms recorded during the experiment. The cardiac rhythms were classified as organized rhythms since running QRS complexes were observed in the ECG waveforms. When the ABP waveforms were examined during compression pause, no identifiable pulse was observed in left panel while spontaneous pulses were immediately visible in right panel, the rhythms were therefore annotated as PEA and PR respectively. CC and hands-off intervals could be easily identified from the large amplitude ACC signals (Fig 1C). But the oscillations during the CC pauses were hardly visible in both cases. When the waveforms were enlarged, only artifacts were observed for PEA while oscillations synchronized with the mechanical contraction of the heart became identifiable in the presence of background noise for PR (red circles in Fig 1D).

Fig 2 shows the spontaneous pulse detection procedure based on cross-correlation function between filtered ECG and ACC signals. After pre-processing, the background artifacts in the original ACC signals (Fig 2D) were successfully suppressed whereas oscillations synchronized with QRS complexes were retained. The morphologic pattern of cross-correlation function for PEA was different from that of PR and the resulted CCp values were 0.048 and 0.701 respectively.

**Fig 3:**
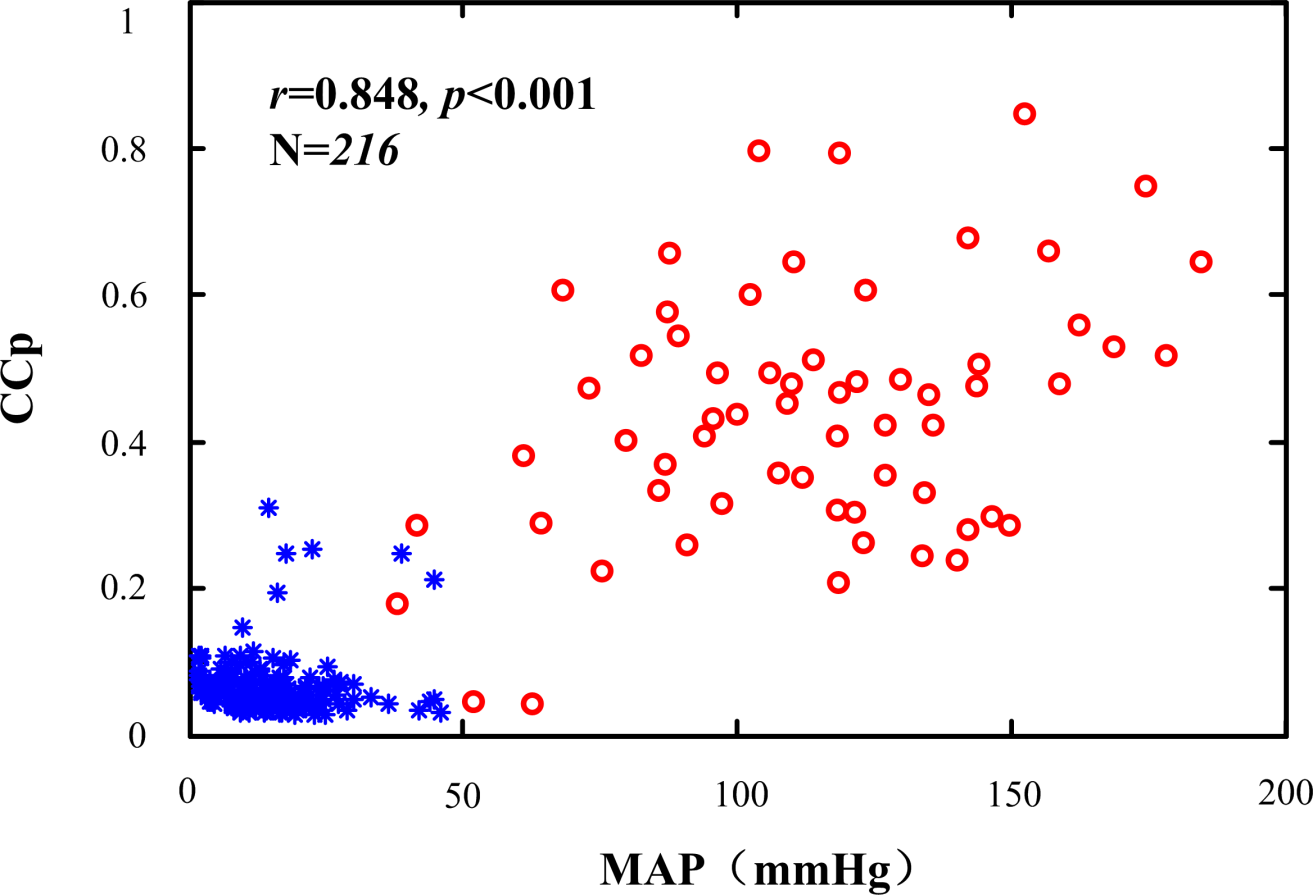
The relationship between peak correlation coefficient (CCp) of cross-correlation function and mean arterial pressure (MAP). * and o represent pulseless electrical activity and perfusing rhythm respectively.

The calculated MAP, CCp and HR are summarized in Table 1. MAP (115.3±36.3 vs. 15.2±9.5 mmHg, *p*<0.001), CCp (0.440±0.176 vs. 0.067±0.042, *p*<0.001) and HR (159±51 vs. 86±45beats/min, *p*<0.001) were significantly higher for PR as compared to PEA. The relationship between CCp and MAP is shown in Fig 3. The Pearson correlation coefficient was 0.848 (*p*<0.001).

**Table 1:** A comparison of mean arterial pressure (MAP), peak correlation coefficient (CCp) and heart rate (HR) between segments of pulseless electrical activity (PEA) and perfusing rhythm (PR) during chest compression pauses.

	MAP (mmHg)	CCp	HR (beats/min)
PEA (N = 153)	15.2±9.5	0.067±0.042	86±45
PR (N = 63)	115.3±36.3	0.440±0.176	159±51
*p* value	<0.001	<0.001	<0.001

A total of 32 PR and 74 PEA were distributed into training set. The respective MAP, CCp, HR value of 49.0, 0.22 and 151 that provided highest accuracy was selected as the optimal threshold to discriminate PR and PEA. The testing set included 31 PR and 79 PEA. The performances of spontaneous pulse detection based on MAP, CCp and HR are listed in Table 2. The discriminative ability of MAP tended to be higher than that of CCp, but was not statistically significant in terms of AUC (0.999 vs. 0.965, *p* = 0.127), sensitivity (96.8% vs. 93.6%, *p* = 0.554), specificity (100% vs. 97.5%, *p* = 0.155) and accuracy (99.1% vs. 96.7%, *p* = 0.175). HR was an inferior discriminator for spontaneous pulse as compared to both CCp and MAP. Specifically, AUC (0.965 vs. 0.894, *p* = 0.038), sensitivity (93.6% vs. 48.4%, *p*<0.001) and accuracy (96.7% vs. 82.7%, *p*<0.001) were significantly higher for CCp than HR, as well as for MAP than HR.

**Table 2:** Performance of mean arterial pressure (MAP), peak correlation coefficient (CCp) and heart rate (HR) for spontaneous pulse detection using testing set (31 perfusing rhythm and 79 pulseless electrical activity).

	AUC	Sensitivity (%)	Specificity (%)	Accuracy (%)
MAP	0.999	96.8	100	99.1
CCp	0.965#	93.6#	97.5	96.7#
HR	0.894*	48.4*	96.2	82.7*

*: *p*<0.05 compared with MAP

#: *p*<0.05 compared with HR.

## Discussion

The present study demonstrated that it is feasible to perform reliable automated spontaneous pulse detection during CC pauses by analyzing ACC signals acquired from accelerometer-based CPR feedback sensor that was placed on the surface of the animal’s chest over the heart. The proposed method would eliminate unnecessary pauses for spontaneous pulse detection because it uses only the timed pauses for ventilation or cardiac rhythm analysis.

Reliable detection of spontaneous pulse with minimal interruptions of CC is part of high-quality CPR. In order to detect the presence or absence of spontaneous pulses, several methods have been attempted by using different transducers and sensors. In a porcine model of cardiac arrest, Wijshoff et al. [29] reported that photoplethysmography (PPG) signals obtained by placing a forehead reflectance pulse oximetry probe between the nostrils allowed for the detection of a spontaneous pulse during ventilation pauses. But the sensitive to motion artifacts of PPG sensor may limit its clinical application. In another animal study, Reynolds et al. [30] demonstrated peripheral oxygen saturation (StO_2_) recorded by placing a near-infrared spectroscopy probe on the left forelimb rapidly decreased after loss of pulses but increased extremely slowly after ROSC. Ruiz [31] and Alonso [32] reported that thoracic impedance signals acquired from defibrillation pads could be used to differentiate PR from PEA rhythms reliably in OHCA patients. However, the thoracic impedance based spontaneous pulse detection method can’t be launched during ventilation pauses because ventilations create much higher oscillations in the thoracic impedance signals than the mechanical contractions of the heart.

The emphasis on high quality CPR by current resuscitation guidelines prompted the evaluation of CPR quality using signals registered by modern AEDs [33]. One of the most commonly used real-time CPR feedback system is based on accelerometer sensors placed either in an external device or incorporated into the defibrillation pads [34, 35]. The clinical relevant CC parameters, including depth, rate, and hands-off intervals can be assessed by the simultaneously recorded ACC waveforms in AEDs [36].

The current study focused on analyzing ACC waveforms recorded during hands-off intervals or ventilation pauses to distinguish PR from PEA. We observed that the movements resulted from mechanical contractions of the perfusing heart could be transferred to the chest wall and captured by the accelerometer-based CPR sensor that placed on the surface of the chest. Unlike the ventilation induced high amplitude oscillations in thoracic impedance signals acquired from defibrillation pads, ACC signals were not affected by the relatively slow movement of the chest wall when ventilation was delivered. Additionally, the spontaneous circulation initiated oscillations in ACC waveforms were aligned with the QRS complexes and blood pressure pulses but different with the background artifacts in amplitude and morphology. After pre-processing, the background artifacts were successfully suppressed and the presence or absence of spontaneous pulses could be detected by the proposed algorithm. The maximal coefficient of cross-correlation between ECG and ACC signals, i.e. CCp was highly correlated with MAP and significantly different between PR and PEA rhythms. The 93.6% sensitivity and 97.5% specificity exceeded the 90% and 95% lower limit recommended by the American Heart Association Task Force on AEDs for accurate detection of VF, which were also suggested by Eberle et al. [5] as the diagnostic accuracy for spontaneous pulse detection. The ability of detection of spontaneous pulses for the accelerometer-based method was closely comparable to the gold standard of invasive blood pressure measurement, and outperformed the HR based method.

The proposed method provides a new option for circulation detection and can be easily incorporated into AEDs as well as into defibrillators with the utilization of accelerometers as real-time CPR feedback. Since the CC induced oscillations are much higher (46.35±15.95 vs. 1.83±0.96 m/s^2^, *p*<0.001) than the mechanical contractions related oscillations in ACC waveforms, the CC pauses can be correctly identified by checking the amplitude of ACC signals. The high reliability of automated rhythm assessment algorithm already allows for accurate organized rhythms detection during CC pauses [37]. If an organized rhythm is identified, the proposed method will further differentiate PEA from PR. For instances of compression-only CPR, spontaneous pulses detection can be performed during the hands-off intervals for rhythm analysis. For instances of conventional 30:2 CPR, circulation detection can be performed both during the CC pauses for ventilation and during the hands-off intervals for rhythm analysis.

There are several limitations to the current study. First, although swine have increasingly been employed in resuscitation and critical care research because they share similar cardiovascular and physiologic properties with humans, the animal model used in this study still cannot fully represent clinical scenarios. Therefore, the results of current study still need to be evaluated in OHCA patients. Second, factors may affect the ACC signals and the ability of spontaneous pulses detection, such as location and contact of ACC sensors, have not been addressed. Third, the proposed method has not been compared with other reported techniques, including PPG, StO_2_ and thoracic impedance based circulation detection. Additionally, it is still challenging to identify the spontaneous pulse during uninterrupted CC.

## Conclusions

In this study, a method to detect the presence of spontaneous pulse has been proposed using the ECG and ACC signals acquired from accelerometer-based CPR feedback sensor during chest compression pauses. The retrospective animal data demonstrated its feasibility as a tool to confirm cardiac arrest and to detect ROSC with high accuracy during resuscitation and CPR.

## Supporting information

S1 File Training and testing data sets. (XLSX)

S2 File ARRIVE checklist. (DOC)
